# The predictive value of lesion-specific pericoronary fat attenuation index for major adverse cardiovascular events in patients with type 2 diabetes

**DOI:** 10.1186/s12933-024-02272-5

**Published:** 2024-06-04

**Authors:** Meiju Liu, Yanhua Zhen, Jin Shang, Yuxue Dang, Qian Zhang, Weishi Ni, Yujuan Qiao, Yang Hou

**Affiliations:** Department of Radiology, Shengjing Hospital of China Medical University, No. 36, Sanhao Street, Heping District, Shenyang, 110004 Liaoning China

**Keywords:** Type 2 diabetes mellitus, Coronary computed tomography angiography, Major adverse cardiovascular events, Fat Attenuation Index

## Abstract

**Background:**

The purpose of this study was to explore the prognostic significance of the lesion-specific pericoronary fat attenuation index (FAI) in forecasting major adverse cardiovascular events (MACE) among patients with type 2 diabetes mellitus (T2DM).

**Methods:**

This study conducted a retrospective analysis of 304 patients diagnosed with T2DM who underwent coronary computed tomography angiography (CCTA) in our hospital from December 2011 to October 2021. All participants were followed for a period exceeding three years. Detailed clinical data and CCTA imaging features were carefully recorded, encompassing lesion-specific pericoronary FAI, FAI of the three prime coronary arteries, features of high-risk plaques, and the coronary artery calcium score (CACS). The MACE included in the study comprised cardiac death, acute coronary syndrome (which encompasses unstable angina pectoris and myocardial infarction), late-phase coronary revascularization procedures, and hospital admissions prompted by heart failure.

**Results:**

Within the three-year follow-up, 76 patients with T2DM suffered from MACE. The lesion-specific pericoronary FAI in patients who experienced MACE was notably higher compared to those without MACE (–84.87 ± 11.36 Hounsfield Units (HU) vs. –88.65 ± 11.89 HU, *p* = 0.016). Multivariate Cox regression analysis revealed that CACS ≥ 100 (hazard ratio [HR] = 4.071, 95% confidence interval [CI] 2.157–7.683, *p* < 0.001) and lesion-specific pericoronary FAI higher than − 83.5 HU (HR = 2.400, 95% CI 1.399–4.120, *p* = 0.001) were independently associated with heightened risk of MACE in patients with T2DM over a three-year period. Kaplan-Meier analysis showed that patients with higher lesion-specific pericoronary FAI were more likely to develop MACE (*p* = 0.0023). Additionally, lesions characterized by higher lesion-specific pericoronary FAI values were found to have a greater proportion of high-risk plaques (*p* = 0.015). Subgroup analysis indicated that lesion-specific pericoronary FAI higher than − 83.5 HU (HR = 2.017, 95% CI 1.143–3.559, *p* = 0.015) was independently correlated with MACE in patients with T2DM who have moderate to severe coronary calcification. Moreover, the combination of CACS ≥ 100 and lesion-specific pericoronary FAI>-83.5 HU significantly enhanced the predictive value of MACE in patients with T2DM within 3 years.

**Conclusions:**

The elevated lesion-specific pericoronary FAI emerged as an independent prognostic factor for MACE in patients with T2DM, inclusive of those with moderate to severe coronary artery calcification. Incorporating lesion-specific pericoronary FAI with the CACS provided incremental predictive power for MACE in patients with T2DM.

## Background

Type 2 diabetes mellitus (T2DM) is a multifaceted metabolic condition that precipitates extensive damage across a variety of tissues, encompassing blood vessels, microvessels, and nerves. Cardiovascular disease emerges as the foremost cause of death among individuals afflicted with T2DM. The incidence of cardiovascular complications in diabetic patients is 2–4 times higher than those without diabetes [1]. Consequently, accurately predicting major adverse cardiovascular events (MACE) in patients with T2DM is of great importance in order to enhance their quality of life and prolong their survival.

The Arteriosclerotic Cardiovascular Disease risk calculator, endorsed by the American Diabetes Association [2] is recognized as a prevalent method for appraising cardiovascular risk in patients with T2DM. This tool incorporates various clinical cardiovascular risk determinants such as age, gender, ethnicity, blood pressure, lipid profiles, history of diabetes, smoking habits, and medication records. However, it is important to note that this calculator is not specifically designed for patients with T2DM and does not account for specific risk factors like diabetes duration and related complications, which may limit its ability to predict MACE in these patients [3]. Despite the integration of diabetes-specific risk markers by some alternative models, the enhancement in predictive accuracy remains relatively incremental when compared to the general population models [4]. Therefore, the development of novel biomarkers is imperative to refine the predictive performance of current models for MACE in patients with T2DM.

Pericoronary adipose tissue (PCAT) has recently emerged as a promising biomarker indicative of coronary artery inflammation. Patients with T2DM typically exhibit insulin resistance and persistent hyperglycemia, which trigger counter-regulatory mechanisms to bolster insulin production. This response is thought to promote the expansion of visceral adipose cells, leading to hypoxia and consequent inflammation in the adipose milieu [5]. As such, the inflammatory status of PCAT is more pronounced in patients with T2DM compared to their non-diabetic patients [6]. Antonopoulos et al. [7] have unveiled a novel non-invasive imaging phenotype, the perivascular adipose tissue fat attenuation index (FAI), aimed at assessing PCAT inflammation. Studies have demonstrated [8–10] that the FAI is instrumental in detecting high-risk plaques, predicting the progression of coronary artery plaques, identifying patients susceptible to forming high-risk plaques, and forecasting patient outcomes. However, its clinical utility in populations with T2DM remains to be further validated.

Although prior studies have indicated a markedly increased FAI in the right coronary artery (RCA) of patients with T2DM relative to non-diabetic subjects [6], evidence validating the utility of RCA-FAI as a harbinger for MACE in the T2DM cohort is still lacking. Scholarly focus is converging on the FAI of the prime coronary arteries in patients with T2DM, yet the impact of lesion stenosis severity on FAI has been overlooked. Addressing this gap, our current study endeavors to determine if lesion-targeted FAI can bolster the prognostic value for MACE in patients with T2DM, thereby facilitating stratified risk alerts for individuals at heightened peril.

## Materials and methods

### Study population

Our study retrospectively analyzed 1,018 patients with T2DM who underwent coronary computed tomography angiography (CCTA) at our hospital between December 2011 and October 2021. The participants ranged in age from 33 to 88 years, and all participants were adults. T2DM was defined according to one or more of the following criteria: the use of oral hypoglycemic agents or insulin therapy; a fasting blood glucose level equal to or exceeding 7.0 mmol/L; or an glycated hemoglobin A1c (HbA1c) level equal to or exceeding 6.5% [11]. During data collection, patients’ medical records were thoroughly examined, including prescription details, laboratory findings, and clinical documentation, to verify the precision of the T2DM diagnosis. Cardiovascular risk factors were categorized based on specific criteria: (a) Hypertension was confirmed either through prior diagnosis, systolic blood pressure exceeding 140 mmHg, or diastolic blood pressure exceeding 90 mmHg; (b) A history of smoking included individuals who previously smoked or were current smokers; (c) Obesity was defined as a body mass index (BMI) of 28 kg/m^2^ or higher according to Expert consensus on obesity prevention and treatment in China [12]. The Framingham Risk Score (FRS) [13], encompassing variables such as age, gender, total cholesterol, high-density lipoprotein, systolic blood pressure, and smoking status, was employed to stratify patients’ coronary heart disease risk into three categories: high risk (≥ 20% within 10 years), intermediate risk (10–19% within 10 years), and low risk (< 10% within 10 years). Triglyceride-glucose (TyG) index = ln[Triglyceride(mg/dl)×Fasting blood glucose(mg/dl)/2]. Systemic inflammatory response index (SIRI) = Neutrophils×Monocyte/Lymphocyte. The study protocol was approved by the Medical Ethics Committee of The Shengjing Hospital of China Medical University (No. 2023PS1048K) and conducted in accordance with the principles contained within the Declaration of Helsinki. All patients enrolled in the study provided informed consent.

**Fig. 1:**
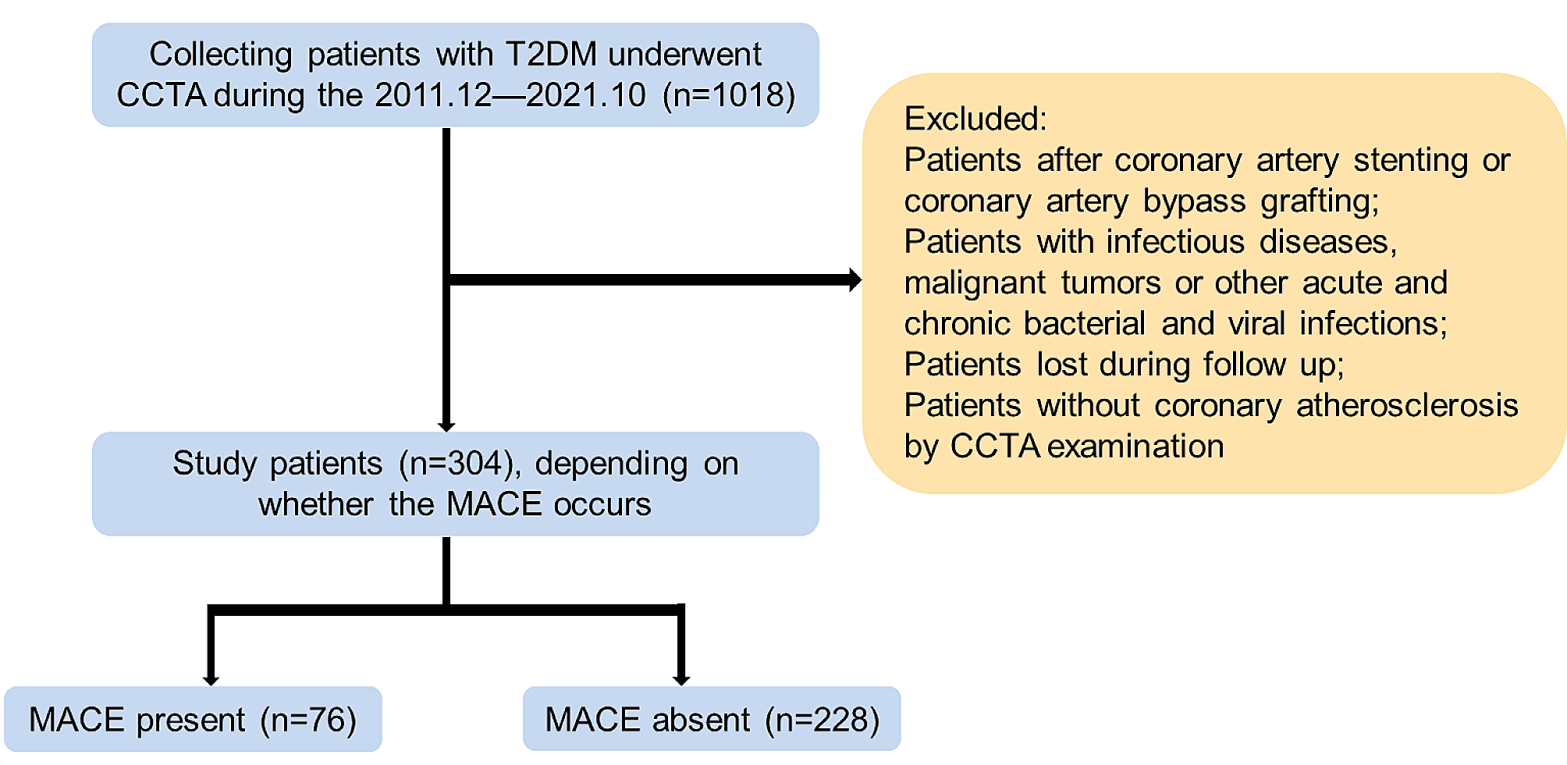
Flowchart showing the study design. *T2DM* type 2 diabetes mellitus, *CCTA* coronary computed tomography angiography, *MACE* major adverse cardiovascular event

The following exclusion criteria were applied to define the study cohort: individuals who had previously received coronary stent placement or coronary artery bypass graft surgery; patients diagnosed with infectious diseases, malignancies, or suffering from acute or chronic bacterial and viral infections; subjects who exhibited no signs of coronary sclerosis as per the CCTA examination findings; and participants who were lost during the follow-up period (Fig. 1).

### Data collection

In our investigation, baseline characteristics of patients with T2DM were meticulously recorded through clinical hospitalization records from the Neusoft Hospital Information System. The data captured included demographics (gender, age), clinical parameters (systolic and diastolic blood pressure, BMI), cardiovascular risk factors, and details concerning laboratory tests and prescribed medications. To gather follow-up information, we conducted telephone interviews and reviewed patients’ details in the hospitalization system. We ensured that all participants were monitored for a minimum of three years following their CCTA. The main outcome we focused on was the incidence of MACE, which comprised a combination of cardiac-related death, acute coronary syndrome (spanning unstable angina and myocardial infarction), late-phase coronary revascularization procedures, and hospitalizations triggered by heart failure.

### CCTA acquisition

The Brilliance iCT (Philips Healthcare, The Netherlands) and Spectral IQon (Philips Healthcare, The Netherlands) CT scanners were used to perform CCTA on patients with T2DM. The scanning range extended from 1.0 cm below the tracheal bifurcation to the cardiac apex. Contrast agent tracking triggering technique was used, with the region of interest set within the ascending aorta at the level of the main pulmonary artery. The triggering threshold was meticulously set at 150 HU. The triggering threshold was meticulously set at 150 HU. Upon reaching this threshold, patients were instructed to hold their breath, and scanning commenced after a delay of 6 s. The contrast agent, either iodixanol or iopromide at a concentration of 350 mg/mL, was administered via an Ulrich REF XD 2051 dual-barrel high-pressure injector. We used an 18G cannula inserted into the elbow vein, ensuring an injection flow rate between 4.0 and 6.0 mL/s and an injection volume calibrated to 0.6–0.8 mL/kg of the patient’s body weight. Scan parameters were meticulously set: for the iCT a tube voltage of 100 or 120 kV, and for the IQon CT always at 120 kV; tube current modulation was applied using prospective gating with a Dose Right Index (DRI) of 13, triggered at the optimal 78% phase of the R-R interval with an exposure and data acquisition buffer zone of ± 3%, and retrospective gating set at a DRI of 28; rotation time was kept swift at 0.27 s; collimation settings for both the iCT at 128 × 0.625 mm and the IQon CT at 64 × 0.625 mm. The field of view was standardized to 250 × 250 mm, with reconstruction detailing a layer thickness of 0.9 mm and an interval of 0.45 mm. In scenarios where a patient’s heart rate was elevated, measured at ≥ 70 beats/min, they were given metoprolol tartrate tablets (ranging from 25 to 50 mg, manufactured by AstraZeneca Pharmaceuticals) prior to scanning to moderate and stabilize the heart rate. Subsequent image sequence reviews meticulously identified the phases offering the utmost image clarity and minimal motion artifacts for further detailed analysis.

### CCTA image analysis

In patients with T2DM who experienced MACE, we selected the criminal plaque for analysis. Conversely, in those T2DM patients without MACE, we selected the plaque exhibiting the most severe stenosis for further examination. The location of criminal plaque was determined by combining electrocardiogram, echocardiography, and coronary angiography (CAG) examinations.

The plaque analysis mainly included three aspects: the degree of lumen stenosis at the plaque, the type of plaque, and high-risk characteristics. The degree of lumen stenosis was classified into five categories: slight stenosis (< 25%), mild stenosis (25–49%), moderate stenosis (50–69%), severe stenosis (70–99%), and occlusion. Plaque types were classified into calcified, non-calcified, and mixed. The characteristics of high-risk plaque in CCTA images were as follows: low-density plaques with CT values below 30 HU and an area greater than 1 mm^2^; positive remodeling indicated by a remodeling index exceeding 1.1; speckled calcifications identified within non-calcified plaques with a long diameter under 3 mm and density above 130 HU; and the napkin-ring sign, denoting a slightly elevated density rimming low-density plaques, with a minimum of two high-risk characteristics required for a plaque to be classified as high-risk [14].

According to the American Heart Association Coronary Artery Segmentation Method, the Gensini score [15] was used to quantitatively evaluate the degree of vascular stenosis for all coronary artery lesions. We defined the Gensini score as follows: a total score of 0 to 30 points represents mild lesions, 31–60 points represents moderate lesions, and scores > 60 points represent severe lesions. The quantification analysis of the Coronary Artery Calcification Score (CACS) [16] was performed using the Philips IntelliSpace Portal workstation (Philips Healthcare) to assess the overall calcification of the coronary arteries. The Agatston method was used for calculation, CACS was categorized as follows: 0, 1–99, 100–399, and ≥ 400, representing no, mild, moderate, and severe calcification, respectively [17]. CT-Fractional Flow Reserve (CT-FFR) analysis was conducted using the Coronary Artery CT Blood Flow Reserve Score Analysis software (Beijing Shukun Network Technology Co., Ltd., China). According to the guidelines of the Radiologist Branch of the Chinese Medical Association [18], lesions with CT-FFR value > 0.80 were considered no ischemia whereas lesions with CT-FFR value ≤ 0.80 was regarded ischemia, CT-FFR value between 0.70 and 0.80 was considered a gray zone.

### Analysis of FAI

**Fig. 2:**
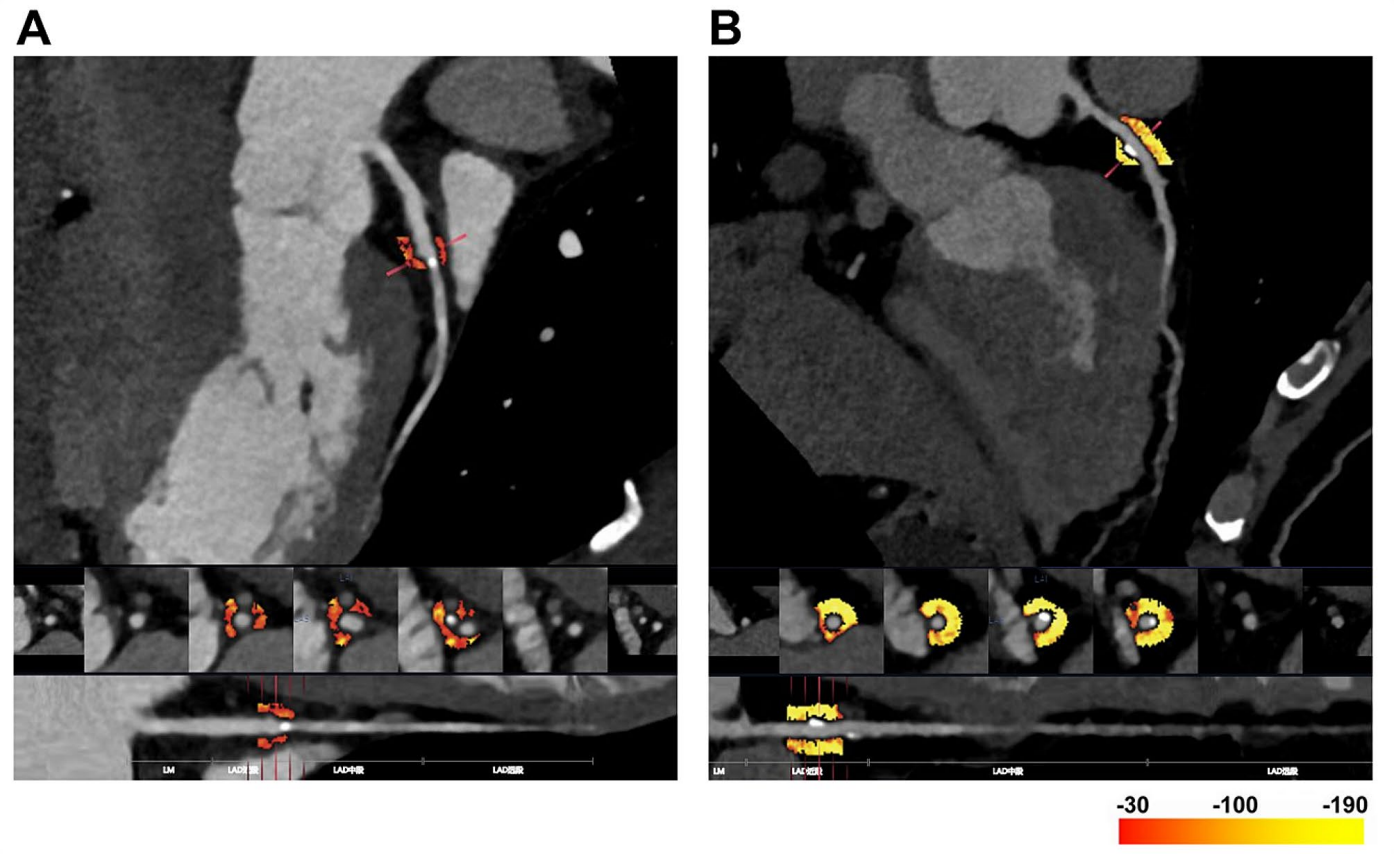
Two typical cases of lesion-specific pericoronary FAI differences between patients with and without MACE measured by CCTA. **A** A 59-year-old patient (male) with MACE, the lesion-specific pericoronary FAI was − 60HU. **B** A 61-year-old patient (male) without MACE, the lesion-specific pericoronary FAI was − 110HU. *FAI* fat attenuation index, *MACE* major adverse cardiovascular event, *CCTA* coronary computed tomography angiography, *HU* Hounsfield Unit

The intelligent analysis software for the Perivascular Adipose Tissue FAI (Beijing Shukun Network Technology Co., Ltd., China) was used to measure the coronary artery FAI and the lesion-specific pericoronary FAI. PCAT was defined as all the voxels between − 190 and − 30 HU range located within a radial distance from the outer vessel wall equal to the average diameter of the target vessel. To measure the FAI of the Left anterior descending (LAD) and Left circumflex (LCX), the range of 0–4 cm from the opening of the LAD and LCX was selected, to measure the FAI of the RCA, the range of 1–5 cm from the opening of the RCA was selected [7, 19]. For lesion-specific pericoronary FAI measurement, the range from 5 mm proximal to the beginning of the plaque to the distal end of the plaque was selected (Fig. 2).

### Statistical analysis

Statistical analysis was performed using R statistical software version 4.2.3. Continuous variables were presented as means and standard deviations and evaluated using independent sample t-tests to assess intergroup differences. Categorical variables were presented as frequencies and percentages and assessed for intergroup differences using chi-square tests. Non-normally distributed data were analyzed using non-parametric tests. The Kaplan–Meier method was used to calculate cumulative survival probability, and the log-rank test was used for comparisons. Univariate Cox regression analysis was conducted to identify potential predictors for the occurrence of MACE in patients with T2DM within three years. Variables with *P* < 0.10 in the univariate analysis were included in the multivariate Cox regression analysis. The results of the multivariate Cox regression analysis were presented as hazard ratios (HR) and 95% confidence intervals (CI).

Receiver operating characteristic (ROC) analysis was used to determine the optimum cutoff values of continuous variables, and the best cutoff values were taken at the maximal Youden index. The predictive value of lesion-specific pericoronary FAI and other risk factors for MACE was evaluated and assessed using ROC curve analysis. Bilateral p values < 0.05 were considered statistically significant.

## Results

### Patient characteristics

A total of 304 patients with T2DM complicated with coronary artery stenosis were included in this study. Patients were divided into two groups according to whether MACE occurred during follow-up. The baseline and imaging features of two groups were shown in Tables 1, 2 and 3. Generally speaking, patients with MACE had a longer duration of diabetes (*p* = 0.05). Lesion-specific pericoronary FAI in patients with MACE was significantly higher than that in patients without MACE (–84.87 ± 11.36 HU vs. –88.65 ± 11.89 HU, *p* = 0.016). In both groups, patients with T2DM who experienced MACE had significantly higher CACS (*p* < 0.001), higher Gensini scores (*p* < 0.001), greater extent of vascular stenosis resulting from culprit plaque (*p* < 0.001), increased non-calcified and mixed plaque (*p* = 0.006), more high-risk plaques (*p* = 0.028), and lower CT-FFR value (*p* = 0.001) of the blood vessel with plaque. However, there was no significant difference in RCA-FAI between two groups (–89.18 ± 10.29 HU vs. –90.21 ± 10.83 HU, *p* = 0.471).

**Table 1:** Comparison of baseline characteristics between patients with T2DM with and without MACE

Parameters	ALL (*n* = 304)	MACE present (*n* = 76)	MACE absent (*n* = 228)	*P*
Sex (male)	157 (51.6%)	45 (59.2%)	112 (49.1%)	0.13
Age	61.79 ± 9.86	63.21 ± 10.39	61.32 ± 9.65	0.15
Systolic pressure (mmHg)	138.15 ± 18.43	139.71 ± 18.22	137.64 ± 18.51	0.40
Diastolic pressure (mmHg)	82.28 ± 10.98	82.72 ± 11.35	82.14 ± 10.88	0.69
BMI (kg/m^2^)	25.17 ± 3.21	24.91 ± 3.01	25.25 ± 3.28	0.43
Obesity	55 (18.1%)	9 (11.8%)	46 (20.2%)	0.10
Hypertension	202 (66.9%)	49 (66.2%)	153 (67.1%)	0.89
Smoke	81 (26.8%)	20 (27.0%)	61 (26.8%)	0.96
Drink	82 (27.2%)	22 (29.7%)	60 (26.3%)	0.57
DM life (year)	5.00 (0.50-10.75)	7.00 (2.00–12.00)	4.00 (0.33-10.00)	0.05*
Blood glucose	7.74 ± 2.83	8.16 ± 2.78	7.60 ± 2.84	0.13
HbA1c (%)	7.33 ± 1.65	7.48 ± 1.74	7.28 ± 1.62	0.35
TG (mmol/L)	2.11 ± 1.51	2.15 ± 1.73	2.10 ± 1.43	0.80
TC (mmol/L)	4.62 ± 1.05	4.58 ± 1.08	4.63 ± 1.05	0.74
HDL (mmol/L)	1.08 ± 0.28	1.10 ± 0.29	1.08 ± 0.28	0.47
LDL (mmol/L)	2.82 ± 0.85	2.78 ± 0.81	2.83 ± 0.86	0.62
WBC (×10^9/L)	6.41 ± 1.62	6.34 ± 1.48	6.43 ± 1.67	0.68
Neu (×10^9/L)	3.80 ± 1.21	3.81 ± 1.18	3.80 ± 1.23	0.99
Lym (×10^9/L)	2.07 ± 1.75	1.93 ± 0.56	2.12 ± 1.99	0.42
Mono (×10^9/L)	0.45 ± 0.15	0.44 ± 0.14	0.45 ± 0.15	0.52
Platelet (×10^9/L)	211.19 ± 57.41	218.12 ± 53.94	208.87 ± 58.46	0.22
Antiplatelet drug	217 (71.4%)	60 (78.9%)	157 (68.9%)	0.09
Statin	236 (77.6%)	58 (76.3%)	178 (78.1%)	0.75
Diuretic	14 (4.6%)	2 (2.6%)	12 (5.3%)	0.34
β-receptor blocker	112 (36.8%)	32 (42.1%)	80 (35.1%)	0.27
CCB	132 (43.4%)	35 (46.1%)	97 (42.5%)	0.59
ACEI/ARB	158 (52.0%)	42 (55.3%)	116 (50.9%)	0.51
Hypogilcemic	217 (71.4%)	51 (67.1%)	166 (72.8%)	0.34
Insulin	94 (30.9%)	26 (34.2%)	68 (29.8%)	0.47
Hypogilcemic + Insulin	82 (27.0%)	23 (30.3%)	59 (25.9%)	0.46
FRS	15.02 ± 3.84	15.03 ± 3.95	15.01 ± 3.82	0.98
FRS risk grade				0.34
Low	142 (46.7%)	30 (39.5%)	112 (49.1%)	
Middle	92 (30.3%)	26 (34.2%)	66 (28.9%)	
High	70 (23.0%)	20 (26.3%)	50 (21.9%)	
TyG index	9.23 ± 0.74	9.27 ± 0.77	9.21 ± 0.73	0.56
SIRI	0.95 ± 0.59	0.94 ± 0.54	0.95 ± 0.61	0.91

Data are presented as mean ± standard deviation or number (%). P values reflect comparisons between patients with and without major adverse cardiovascular events

*T2DM* type 2 diabetes mellitus, *MACE* major adverse cardiovascular events, *BMI* body mass index, *DM* diabetes mellitus, *HbA1c* glycated hemoglobin A1c, *TG* triglyceride, *TC* total cholesterol, *HDL* high density lipoprotein, *LDL* low density lipoprotein, *WBC* white blood cell, *Neu* neutrophils, *Lym* lymphocyte, *Mono* monocyte, *CCB* calcium channel blockers, *ACEI* angiotensin-converting enzyme inhibitor, *ARB* angiotensin-receptor blocker, *FRS* framingham risk score, *TyG* triglyceride-glucose, *SIRI* systemic inflammatory response index

*Data are means with a statistical difference

**Table 2:** Comparison of lesion plaque imaging characteristics between patients with T2DM with and without MACE

Parameters	ALL (*n* = 304)	MACE present (*n* = 76)	MACE absent (*n* = 228)	*P*
Stenosis degree of lesion plaque vessel				< 0.001*
1–25%	76 (25.0%)	6 (7.9%)	66 (30.7%)	
26–50%	125 (41.1%)	25 (32.9%)	100 (43.9%)	
51–75%	75 (24.7%)	29 (38.2%)	46 (20.2%)	
76–90%	24 (7.9%)	12 (15.8%)	12 (5.3%)	
91–100%	4 (1.3%)	4 (5.3%)	0 (0%)	
Coronary artery of lesion plaque				0.320
LM	5 (1.6%)	2 (2.6%)	3 (1.3%)	
LAD	204 (67.1%)	46 (60.5%)	158 (69.3%)	
LCX	31 (10.2%)	12 (15.8%)	19 (8.3%)	
RCA	63 (20.7%)	16 (21.1%)	47 (20.6%)	
RI	1 (0.3%)	0 (0%)	1 (0.4%)	
Plaque type				0.006*
Calcified	130 (42.8%)	26 (34.2%)	104 (45.6%)	
Non-calcified	93 (30.6%)	19 (25.0%)	74 (32.5%)	
Mixed	81 (26.6%)	31 (40.8%)	50 (21.9%)	
Lesion-specific pericoronary FAI	−87.70 ± 11.85	−84.87 ± 11.36	-88.65 ± 11.89	0.016*
CT-FFR				0.001*
> 0.8	211 (69.4%)	40 (52.6%)	171 (75.0%)	
0.7–0.8	60 (19.7%)	22 (28.9%)	38 (16.7%)	
< 0.7	33 (10.9%)	14 (18.4%)	19 (8.3%)	
High risk plaque	38 (12.5%)	15 (19.7%)	23 (10.1%)	0.028*

*T2DM* type 2 diabetes mellitus, *MACE* major adverse cardiovascular events, *LM* left main coronary artery, *LAD* left anterior descending, *LCX* left circumflex, *RCA* right coronary artery, *RI* ramus intermediate brabch, *FAI* fat attenuation index, *CT-FFR* CT-fractional flow reserve

*Data are means with a statistical difference

**Table 3:** Comparison of baseline imaging characteristics between patients with T2DM with and without MACE

Parameters	ALL (*n* = 304)	MACE present (*n* = 76)	MACE absent (*n* = 228)	*P*
LAD FAI (HU)	–88.54 ± 9.55	–88.79 ± 8.62	–88.45 ± 9.86	0.790
LCX FAI (HU)	–84.44 ± 9.24	–83.67 ± 8.77	–84.70 ± 9.40	0.403
RCA FAI (HU)	–89.95 ± 10.69	–89.18 ± 10.29	–90.21 ± 10.83	0.471
CACS				< 0.001*
0	56 (19.2%)	8 (11.0%)	48 (21.9%)	
1–99	127 (43.5%)	16 (21.9%)	111 (50.7%)	
100–399	64 (21.9%)	25 (34.2%)	39 (17.8%)	
400–1000	30 (10.3%)	14 (19.2%)	16 (7.3%)	
> 1000	15 (5.1%)	10 (13.7%)	5 (2.3%)	
Gensini score				< 0.001*
0–30	269 (88.5%)	57 (75.0%)	212 (93.0%)	
31–60	29 (9.5%)	15 (19.7%)	14 (6.1%)	
> 60	6 (2.0%)	4 (5.3%)	2 (0.9%)	

*T2DM* type 2 diabetes mellitus, *MACE* major adverse cardiovascular events, *LAD* left anterior descending, *LCX* left circumflex, *RCA* right coronary artery, *FAI* fat attenuation index, *HU* Hounsfield units, *CACS* coronary artery calcium score

*Data are means with a statistical difference

### Relationship between lesion-specific pericoronary FAI and MACE in patients with T2DM

**Fig. 3:**
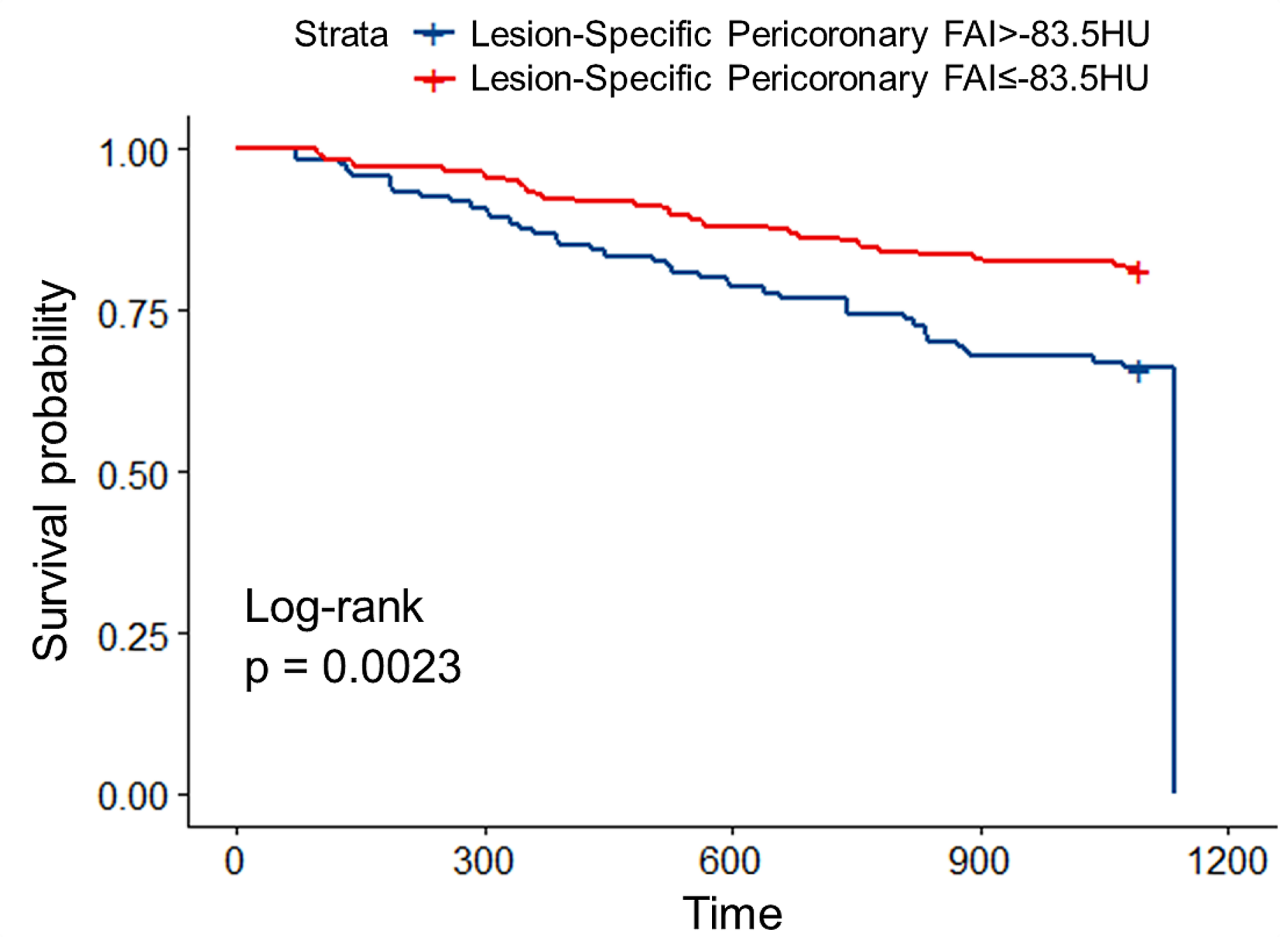
Kaplan–Meier curves of cumulative incidence of major adverse cardiovascular events. Kaplan–Meier curves according to lesion-specific pericoronary fat attenuation index in patients with type 2 diabetes mellitus. *FAI* fat attenuation index

Patients with T2DM who experienced MACE included 53 cases of acute coronary syndrome, 1 case of heart failure, and 22 cases of late coronary revascularisation. Kaplan–Meier analysis showed that during the follow-up time, patients with higher lesion-specific pericoronary FAI experienced a higher incidence of MACE compared to those with lower lesion-specific pericoronary FAI (*p* = 0.0023, log-rank test) (Fig. 3). The univariate Cox regression analysis showed that lesion-specific pericoronary FAI >–83.5 HU, LCX-FAI >–76.5HU, CACS ≥ 100, Gensini score > 60, CT-FFR < 0.70, severe stenosis of vascular lumen caused by criminal plaque, types of plaque and high-risk plaque were all significantly associated with MACE (*p* < 0.10). Furthermore, the multivariate Cox regression analysis identified that both CACS ≥ 100 (HR = 4.071, 95% CI 2.157–7.683, *p* < 0.001) and lesion-specific pericoronary FAI >–83.5 HU (HR = 2.400, 95% CI 1.399–4.120, *p* = 0.001) were independent predictors of MACE (Table 4).

**Table 4:** Risk factors associates with MACE in patients with T2DM

HR	Univariate			Multivariate		
	HR	95% CI	*P*	HR	95% CI	*P*
Obesity	0.567	0.282–1.137	0.110			
DM life	1.308	0.826–2.071	0.253			
HbA1c	1.593	0.840–3.020	0.154			
TyG index	1.253	0.779–2.017	0.352			
SIRI	1.166	0.662–2.052	0.596			
FRS risk grade						
Low	1 (reference)		0.393			
Middle	1.322	0.778–2.248	0.302			
High	1.435	0.815–2.526	0.211			
CACS	4.488	2.732–7.375	< 0.001*	4.071	2.157–7.683	< 0.001*
Gensini score						
Low	1 (reference)		< 0.001*	1 (reference)		0.835
Middle	3.169	1.790–5.609	< 0.001*	1.181	0.558–2.502	0.663
High	4.709	1.706–12.999	0.003*	1.463	0.383–5.582	0.578
CT-FFR						
> 0.8	1 (reference)		0.001*	1 (reference)		0.994
0.7–0.8	2.072	1.222–3.515	0.007*	1.010	0.566–1.802	0.973
< 0.7	2.865	1.558–5.268	0.001*	0.969	0.445–2.107	0.936
LAD FAI	1.481	0.680–3.225	0.322			
LCX FAI	1.582	0.955–2.619	0.075*	0.780	0.425–1.431	0.422
RCA FAI	1.387	0.849–2.266	0.191			
Lesion-specific pericoronary FAI	2.003	1.271–3.156	0.003*	2.400	1.399–4.120	0.001*
Degree of stenosis	3.702	2.128–6.439	< 0.001*	2.142	0.983–4.669	0.055
Plaque type						
Calcified	1 (reference)		0.004*	1 (reference)		0.672
Non-calcified	0.936	0.513–1.706	0.828	1.308	0.680–2.516	0.421
Mixed	2.125	1.261–3.570	0.005*	1.245	0.672–2.308	0.486
High risk plaque	1.991	1.130–3.508	0.017*	1.150	0.594–2.227	0.679

The risk factors were diabetes mellitus life with more than 10 years, lesion-specific pericoronary FAI> – 83.5HU, left anterior descending artery FAI>–98.5HU, left circumflex artery FAI> – 76.5HU, right coronary artery FAI> – 93.5HU, CT-FFR < 0.70, HbA1c > 6.15%, TyG index > 9.02, SIRI > 0.55. The risk factors included severe lumen stenosis, moderate and severe coronary artery calcification (CACS ≥ 100), total Gensini score > 60, non-calcified or mixed plaque in criminal plaque and high risk plaque in criminal plaque

*T2DM* type 2 diabetes mellitus, *MACE* major adverse cardiovascular events, *HR* hazard ratio, *CI* confidence interval, *DM* diabetes mellitus, *HbA1c* glycated hemoglobin A1c, *TyG* triglyceride-glucose, *SIRI* systemic inflammatory response index, *FRS* framingham risk score, *CACS* coronary artery calcium score, *CT-FFR* CT-fractional flow reserve, *LAD* left anterior descending, *LCX* left circumflex, *RCA* right coronary artery, *FAI* fat attenuation index

*Data are means with a statistical difference

We conducted a detailed analysis of the characteristics of lesion plaques in patients with T2DM based on different values of the lesion-specific pericoronary FAI. In 304 lesion plaques, the lesion-specific pericoronary FAI in 121 plaques exceeded − 83.5 HU, while the lesion-specific pericoronary FAI in 183 plaques was equal to or lower than − 83.5 HU. Notably, those lesions with higher lesion-specific pericoronary FAI values were more likely to be high-risk plaques (*p* = 0.015) (Table 5).

**Table 5:** Comparison of lesion plaques imaging characteristics between plaques with higher lesion-specific pericoronary FAI and low lesion-specific pericoronary FAI

Parameters	ALL (*n* = 304)	Lesion-specific pericoronary FAI>– 83.5HU (*n* = 121)	Lesion-specific pericoronary FAI≤– 83.5HU (*n* = 183)	*P*
Stenosis degree of lesion plaque vessel				0.804
Mild stenosis	201 (66.1%)	79 (65.3%)	122 (66.7%)	
Moderate-severe stenosis	103 (33.9%)	42 (34.7%)	61 (33.3%)	
Plaque type				0.149
Calcified	130 (42.8%)	44 (36.4%)	86 (47.0%)	
Non-calcified	93 (30.6%)	39 (32.2%)	54 (29.5%)	
Mixed	81 (26.6%)	38 (31.4%)	43 (23.5%)	
High risk plaque				0.015*
Present	38 (12.5%)	22 (18.2%)	16 (8.7%)	
Absent	266 (87.5%)	99 (81.8%)	167 (91.3%)	

*FAI* fat attenuation index

*Data are means with a statistical difference

**Fig. 4:**
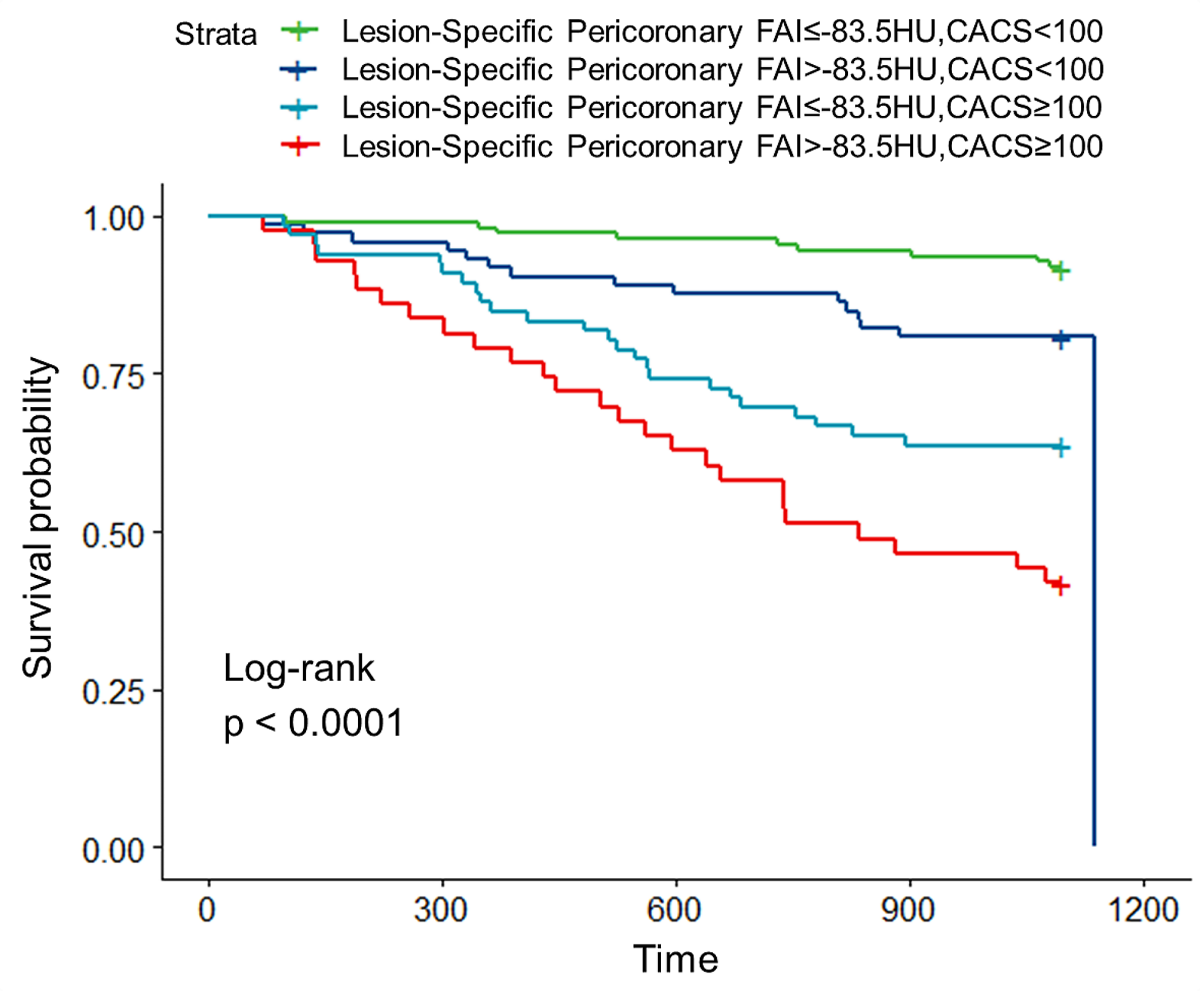
Kaplan–Meier curves of cumulative incidence of major adverse cardiovascular events. Kaplan–Meier curves according to lesion-specific pericoronary fat attenuation index and coronary artery calcium score in patients with type 2 diabetes mellitus. *FAI* fat attenuation index, *CACS* coronary artery calcium score

**Fig. 5:**
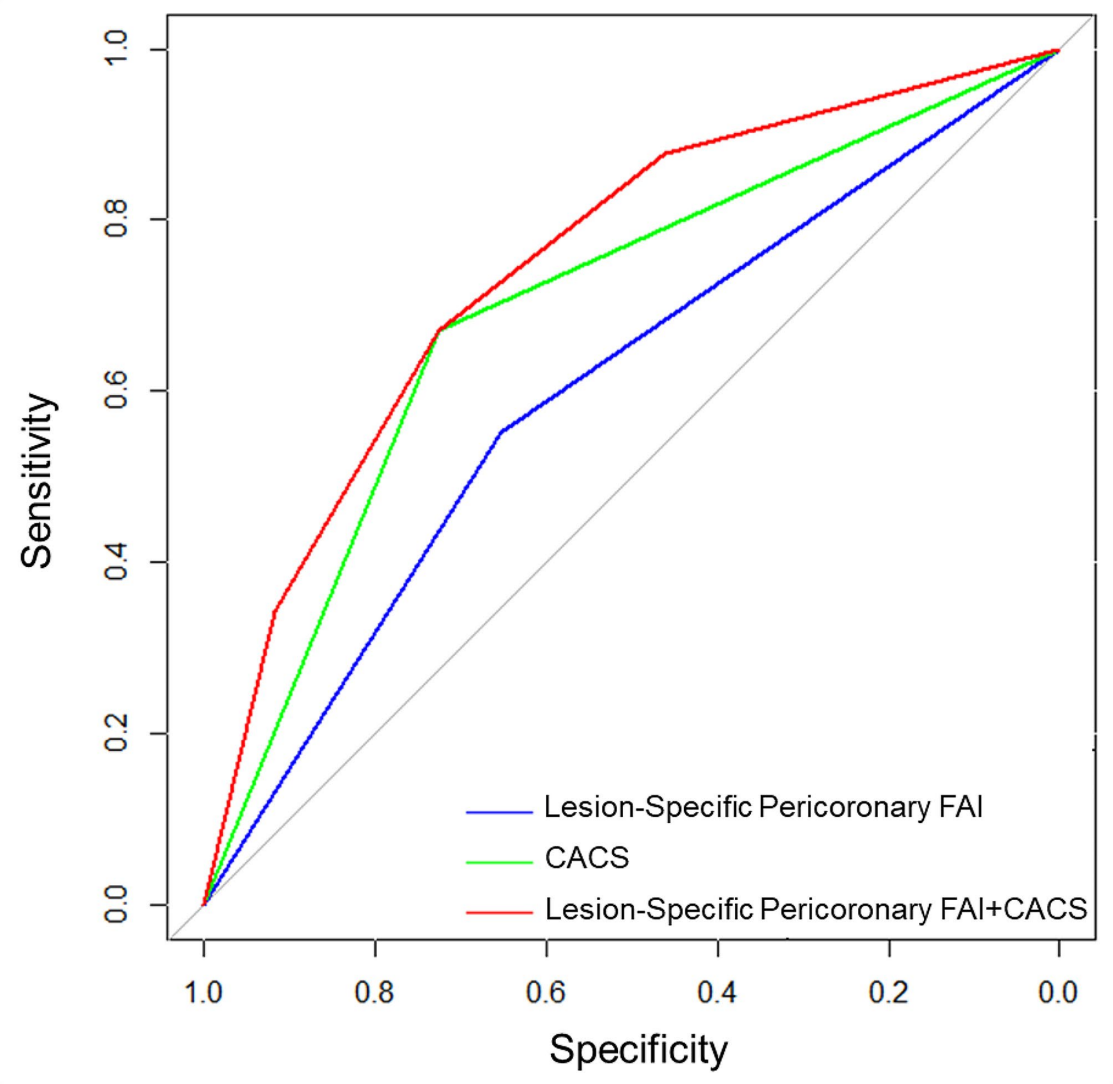
The incremental prognostic value of lesion-specific pericoronary fat attenuation index as compared with CACS for predicting major adverse cardiovascular events. Receiver operating characteristic curves. *FAI* fat attenuation index, *CACS* coronary artery calcium score

Kaplan–Meier analysis showed that during the follow-up time, patients with higher lesion-specific pericoronary FAI who had moderate and severe coronary artery calcification (FAI >– 83.5HU, CACS ≥ 100) experienced a higher incidence of MACE compared to others (*p* < 0.0001, log-rank test) (Fig. 4). Compared with the single factor prediction of MACE by CACS, the area under curve (AUC) increased significantly from 0.699 (95% CI 0.627–0.770) to 0.749 (95% CI 0.684–0.814) (*p* < 0.0 L) after the addition of lesion-specific pericoronary FAI. The combination of CACS ≥ 100 and lesion-specific pericoronary FAI >–83.5 HU significantly enhanced the predictive value for MACE within 3 years in patients with T2DM (as illustrated in Fig. 5).

### Association between lesion-specific pericoronary FAI and MACE in patients with T2DM exhibiting moderate to severe coronary calcification

We further investigated the impact of lesion-specific pericoronary FAI on the occurrence of MACE in patients with T2DM who have moderate to severe coronary artery calcification. During the follow-up period, 49 patients experienced MACE. Patients with T2DM who had higher lesion-specific pericoronary FAI (>– 83.5 HU) experienced a greater number of MACE compared to patients with lower lesion-specific pericoronary FAI (*p* = 0.03). In the multivariate Cox regression analysis, lesion-specific pericoronary FAI >– 83.5 HU (HR = 2.017, 95% CI 1.143–3.559, *p* = 0.015) and severe luminal narrowing of vascular lumen due to criminal plaque (HR = 2.360, 95% CI 1.258–4.426, *p* = 0.007) were found to be independently associated with MACE (Table 6).

**Table 6:** Factors associates with MACE in patients with T2DM with moderate and severe coronary artery calcification (CACS ≥ 100)

HR	Univariate			Multivariate		
	HR	95%CI	*P*	HR	95%CI	*P*
Obesity	0.467	0.185–1.178	0.107			
DM life	0.954	0.541–1.679	0.869			
HbA1c	1.270	0.595–2.710	0.536			
TyG index	1.025	0.580–1.813	0.931			
SIRI	1.356	0.636–2.893	0.431			
FRS risk grade						
Low	1 (reference)		0.985			
Middle	1.003	0.507–1.985	0.994			
High	0.950	0.475–1.899	0.884			
Gensini score						
Low	1 (reference)		0.244			
Middle	1.460	0.766–2.783	0.250			
High	2.113	0.747–5.979	0.158			
CT-FFR						
> 0.8	1 (reference)		0.849			
0.7–0.8	1.084	0.574–2.047	0.804			
< 0.7	1.242	0.591–2.610	0.568			
LAD FAI	1.374	0.545–3.465	0.501			
LCX FAI	1.025	0.564–1.861	0.937			
RCA FAI	0.778	0.432–1.401	0.402			
Lesion-specific pericoronary FAI	1.842	1.052–3.228	0.033*	2.017	1.143–3.559	0.015*
Degree of stenosis	2.120	1.140–3.944	0.018*	2.360	1.258–4.426	0.007*
Plaque type						
Calcified	1 (reference)		0.727			
Non-calcified	0.975	0.364–2.611	0.959			
Mixed	1.249	0.688–2.269	0.465			
High risk plaque	0.891	0.433–1.837	0.756			

The risk factors were diabetes mellitus life with more than 10 years, lesion-specific pericoronary FAI >–83.5HU, left anterior descending artery FAI >–98.5HU, left circumflex artery FAI >–76.5HU, right coronary artery FAI >–93.5HU, CT-FFR < 0.70, HbA1c > 6.15%, TyG index > 9.02, SIRI > 0.55. The risk factors included severe lumen stenosis, total Gensini score > 60, non-calcified or mixed plaque in criminal plaque and high risk plaque in criminal plaque

*T2DM* type 2 diabetes mellitus, *MACE* major adverse cardiovascular events, *CACS* coronary artery calcium score, *HR* hazard ratio, *CI* confidence interval, *DM* diabetes mellitus, *HbA1c* glycated hemoglobin A1c, *TyG* triglyceride-glucose, *SIRI* systemic inflammatory response index, *FRS* framingham risk score, *CT-FFR* CT-fractional flow reserve, *LAD* left anterior descending, *LCX* left circumflex, *RCA* right coronary artery, *FAI* fat attenuation index

*Data are means with a statistical difference

## Discussion

This study investigated the association between lesion-specific pericoronary FAI and MACE in patients with T2DM. Over a monitoring duration of three years, the lesion-specific pericoronary FAI emerged as an independent prognostic marker for MACE in patients with T2DM. Additionally, incorporating lesion-specific pericoronary FAI with CACS enhances the predictive accuracy for MACE among this demographic. To our knowledge, our study is at the forefront of unraveling the significance of lesion-specific pericoronary FAI in foretelling the risk of MACE in patients with T2DM.

PCAT, ectopic adipose tissue around coronary artery, is increasingly recognized for its significant influence in escalating cardiovascular disease risk. As a dynamic endocrine and paracrine organ, the inflammatory reaction of PCAT is usually aggravated, marked by an intensified release of pro-inflammatory mediators in patients with T2DM [20]. These mediators readily permeate the coronary artery walls, undermining endothelial function and expediting atherogenesis [21]—paving the way to complications such as coronary artery constriction, myocardial ischemia, and infarction. Such perturbations in the inflammatory milieu and adipose equilibrium within PCAT are detectable via imaging, revealing a heightened CT attenuation signal [7]. Through this study, we have corroborated the association between altered PCAT attenuation and the progression of T2DM, highlighting its implications for patient prognosis.

RCA-FAI has been regarded as a valuable marker for early detection of inflammation in coronary artery [7]. It serves a dual role, not only mirroring the progression of cardiovascular disease in patients but also providing prognostic information regarding potential future cardiac events in those with coronary heart disease [19, 22]. Previous studies have established that RCA-FAI was markedly elevated in patients with T2DM as opposed to those without, and this holds true irrespective of the degree of arterial stenosis or the vulnerability of plaque formations [6]. Further investigation focusing on individuals with T2DM has highlighted that the effectiveness of RCA-FAI in identifying coronary artery disease was more representative than the LAD and LCX arteries, making it a better diagnostic tool for coronary artery disease in patients with T2DM [23]. Additionally, RCA-FAI has been identified as an independent prognostic factor for the onset of microvascular complications in patients with T2DM [24]. Despite these findings showed that RCA-FAI seems to better reflect the overall inflammatory state of patients with T2DM, our study did not corroborate a statistical discrepancy in RCA-FAI between patients with T2DM who suffered from MACE and those without. This discrepancy may be attributable to a lower presence of criminal plaque in the RCA of patients with T2DM who experienced MACE in this study, compared to patients with T2DM who did not encounter such events.

To date, the use of PCAT-FAI as a prognostic tool for MACE in patients with T2DM has not been extensively explored. The study conducted by Ichikawa et al. showed that higher FAI of LAD-PCAT can significantly predict cardiovascular events in patients with T2DM, whereas RCA-PCAT attenuation cannot [25]. Although vascular-based measurements provide a broad perspective on disease state and risk at an individual patient level, lesion-specific pericoronary measurements may be of particular concern when trying to identify plaque at risk of rupture. Therefore, our study was different from the previous research on PCAT around three coronary arteries, but focused on PCAT around lesion, which may potentially lead to unconscionable MACE in patients with T2DM. In our study, we confirmed that the proportion of high-risk plaques was higher in those lesions with higher lesion-specific pericoronary FAI. However, no significant disparities were observed in either type of plaques or the degree of lumen stenosis. The lesion-specific pericoronary FAI could effectively predict MACE within three years in patients with T2DM and offered a non-invasive imaging marker to predict adverse cardiovascular outcomes. This further demonstrates that the lesion-specific pericoronary FAI is a more sensitive index, offering a more direct insight into the early inflammatory state of possible adverse lesions, which is helpful to better predict the outcome of lesion development.

The incidence of vascular calcification is significantly higher in patients with T2DM compared to non-diabetic individuals. T2DM augments the CACS, thereby elevating the lifetime risk of cardiovascular diseases [26, 27]. Our study further validates the effectiveness of CACS in predicting MACE in patients with T2DM. Nevertheless, coronary artery calcification is an irreversible process that does not regress with appropriate medical therapy (such as statins) and may even progress, thereby limiting its value in secondary prevention. Notably, patients with T2DM tend to have a higher proportion of non-calcified plaques. These plaques are more susceptible to rupture compared to calcified plaques, potentially leading to thrombus formation and acute coronary syndromes. Additionally, non-calcified plaques in patients with T2DM often demonstrate significant inflammation in the arterial wall. Therefore, our study introduces a new metric, lesion-specific pericoronary FAI, to reflect the state of arterial wall inflammation in the target lesion areas of patients with T2DM and to predict MACE. This approach offers a new biomarker for improving the stratification of cardiovascular risk in patients with T2DM.

While an elevated CACS indicates an overall burden of coronary atherosclerosis, it does not provide details on local plaque characteristics. Lesion-specific pericoronary FAI, by focusing on specific lesions rather than the entire coronary artery, provides a more detailed assessment of plaque vulnerability. The combination of these two factors can enhance the predictive power for the occurrence of MACE in T2DM patients, enabling the early identification of high-risk patients for targeted intervention and treatment. For example, patients with higher CACS due to extensive calcified plaques may consider high-intensity statin therapy [28]. While in patients with higher lesion-specific pericoronary FAI, intensifying anti-inflammatory treatment may potentially reduce the occurrence of MACE [7, 29]. Additionally, PCAT can serve as a dynamic biomarker for monitoring the efficacy of drugs like colchicine and semaglutide [30, 31]. By dynamically assessing the changes in MACE risk stratification in T2DM patients, this might herald new opportunities for precision management of patients with T2DM and concurrent coronary heart disease. However, whether the lesion-specific FAI can serve as a guide for future clinical trials and aid in assessing the targeted therapeutic efficacy of modern anti-inflammatory pharmaceuticals necessitates further validation through extensive clinical research endeavors.

Our study, despite its contributions, is not without limitations. Initially, the fact that it is a single-center retrospective inquiry means that the sample size is inherently limited, which may impinge upon the generalizability of our findings. Secondly, recognizing that FAI values can vary across different segments of coronary arteries, it is imperative that future investigations delve into the nuanced relationships between segment-specific pericoronary FAI and the incidence of MACE [32]. Lastly, our study focus on measuring lesion-specific pericoronary FAI exclusively at the outset. The variable treatment regimens that patients underwent throughout the follow-up period could exert an influence on the eventual occurrence of MACE, carrying the potential to skew the outcomes of our research. Hence, it is essential to account for these varying therapeutic approaches when interpreting the results. In summary, while our study provides preliminary evidence for the potential additional value of lesion-specific pericoronary FAI in predicting MACE in patients with T2DM, it must be acknowledged that further research is needed to substantiate these findings. Future studies should include large-scale, multicenter patient cohorts and long-term follow-up to more comprehensively assess the independent predictive ability of lesion-specific pericoronary FAI for MACE.

The findings of our study lead us to several pertinent conclusions. First and foremost, we observed a strong association between elevated lesion-specific pericoronary FAI and the occurrence of MACE among patients with T2DM, extending also to those with moderate to severe coronary artery calcification over a three-year span. Second, integrating lesion-specific pericoronary FAI with CACS markedly improved the prediction of MACE within the patients with T2DM cohort. This combined approach shows promise for the more accurate identification of patients at high risk, thereby enabling more proactive and targeted interventions and treatments for those individuals.

## Conclusions

Our study demonstrated that the CCTA-based elevated lesion-specific pericoronary FAI emerged as an independent prognostic factor for MACE in individuals with T2DM, inclusive of those with moderate to severe coronary artery calcification. Incorporating lesion-specific pericoronary FAI with the CACS provided incremental predictive power for MACE in the patients with T2DM. Our findings may help clinicians identify high-risk patients with T2DM for future MACE, facilitate clinical decision-making and formulate therapeutic strategies.

## Data Availability

No datasets were generated or analysed during the current study. The datasets used and/or analysed during the current study are available from the corresponding author on reasonable request.

## Abbreviations

ACEI: Angiotensin-converting enzyme inhibitor
ARB: Angiotensin-receptor blocker
AUC: Area under curve
BMI: Body mass index
CACS: Coronary artery calcium score
CAG: Coronary angiography
CCB: Calcium channel blockers
CCTA: Coronary computed tomography angiography
CI: Confidence interval
CT-FFR: CT-fractional flow reserve
DM: Diabetes mellitus
DRI: Dose right index
FAI: Fat attenuation index
FRS: Framingham risk score
HbA1c: Glycated hemoglobin A1c
HDL: High density lipoprotein
HR: Hazard ratio
HU: Hounsfield units
LAD: Left anterior descending
LCX: Left circumflex
LDL: Low density lipoprotein
LM: Left main coronary artery
Lym: Lymphocyte
MACE: Major adverse cardiovascular events
Mono: Monocyte
Neu: Neutrophils
PCAT: Pericoronary adipose tissue
RCA: Right coronary artery
RI: Ramus intermediate brabch
ROC: Receiver operating characteristic
SIRI: Systemic inflammatory response index
T2DM: Type 2 diabetes mellitus
TC: Total cholesterol
TG: Triglyceride
TyG: Triglyceride-glucose
WBC: White blood cell

## References

[1] Rawshani A, Rawshani A, Franzén S, Eliasson B, Svensson AM, Miftaraj M, McGuire DK, Sattar N, Rosengren A, Gudbjörnsdottir S (2017). Mortality and Cardiovascular Disease in Type 1 and type 2 diabetes. N Engl J Med.

[2] ElSayed NA, Aleppo G, Aroda VR, Bannuru RR, Brown FM, Bruemmer D, Collins BS, Das SR, Hilliard ME, Isaacs D (2023). 10. Cardiovascular Disease and Risk Management: standards of Care in Diabetes-2023. Diabetes Care.

[3] Wong ND, Sattar N (2023). Cardiovascular risk in diabetes mellitus: epidemiology, assessment and prevention. Nat Rev Cardiol.

[4] Chowdhury MZI, Yeasmin F, Rabi DM, Ronksley PE, Turin TC (2019). Prognostic tools for cardiovascular disease in patients with type 2 diabetes: a systematic review and meta-analysis of C-statistics. J Diabetes Complications.

[5] Pedersen DJ, Guilherme A, Danai LV, Heyda L, Matevossian A, Cohen J, Nicoloro SM, Straubhaar J, Noh HL, Jung D (2015). A major role of insulin in promoting obesity-associated adipose tissue inflammation. Mol Metab.

[6] Yu Y, Ding X, Yu L, Dai X, Wang Y, Zhang J (2022). Increased coronary pericoronary adipose tissue attenuation in diabetic patients compared to non-diabetic controls: a propensity score matching analysis. J Cardiovasc Comput Tomogr.

[7] Antonopoulos AS, Sanna F, Sabharwal N, Thomas S, Oikonomou EK, Herdman L, Margaritis M, Shirodaria C, Kampoli AM, Akoumianakis I (2017). Detecting human coronary inflammation by imaging perivascular fat. Sci Transl Med.

[8] Chinese Association of Radiologists (2024). Chinese expert consensus on the examination and diagnosis of CT for coronary atherosclerotic heart disease. Chin J Radiol.

[9] Antoniades C, Kotanidis CP, Berman DS (2019). State-of-the-art review article. Atherosclerosis affecting fat: what can we learn by imaging perivascular adipose tissue?. J Cardiovasc Comput Tomogr.

[10] Mátyás BB, Benedek I, Raț N, Blîndu E, Parajkó Z, Mihăilă T, Benedek T (2024). Assessing the impact of long-term high-dose statin treatment on Pericoronary inflammation and plaque Distribution-A comprehensive coronary CTA Follow-Up study. Int J Mol Sci.

[11] Ahmad E, Lim S, Lamptey R, Webb DR, Davies MJ (2022). Type 2 diabetes. Lancet.

[12] Chinese Nutrition Society Obesity Prevention and Control Section, Chinese Nutrition Society Clinical Nutrition Section (2022). Expert Consensus on obesity Prevention and Treatment in China[J]. Chin J Epidemiol.

[13] Sullivan LM, Massaro JM, D’Agostino RB (2004). Presentation of multivariate data for clinical use: the Framingham Study risk score functions. Stat Med.

[14] Shaw LJ, Blankstein R, Bax JJ, Ferencik M, Bittencourt MS, Min JK, Berman DS, Leipsic J, Villines TC, Dey D (2021). Society of Cardiovascular Computed Tomography / North American Society of Cardiovascular Imaging - Expert Consensus Document on coronary CT imaging of atherosclerotic plaque. J Cardiovasc Comput Tomogr.

[15] Sinning C, Lillpopp L, Appelbaum S, Ojeda F, Zeller T, Schnabel R, Lubos E, Jagodzinski A, Keller T, Munzel T (2013). Angiographic score assessment improves cardiovascular risk prediction: the clinical value of SYNTAX and Gensini application. Clin Res Cardiol.

[16] Agatston AS, Janowitz WR, Hildner FJ, Zusmer NR, Viamonte M, Detrano R (1990). Quantification of coronary artery calcium using ultrafast computed tomography. J Am Coll Cardiol.

[17] Greenland P, Blaha MJ, Budoff MJ, Erbel R, Watson KE (2018). Coronary calcium score and Cardiovascular Risk. J Am Coll Cardiol.

[18] Quality Control and Safety Management Committee of Radiology Committee of Chinese Medical Association, Intelligence Imaging and Quality Safety Committee of Radiology Committee of Jiangsu Medical Association (2020). Chinese experts consensus on coronary artery CT-derived fractional flow reserve. Chin J Radiol.

[19] Oikonomou EK, Marwan M, Desai MY, Mancio J, Alashi A, Hutt Centeno E, Thomas S, Herdman L, Kotanidis CP, Thomas KE (2018). Non-invasive detection of coronary inflammation using computed tomography and prediction of residual cardiovascular risk (the CRISP CT study): a post-hoc analysis of prospective outcome data. Lancet.

[20] Liu Y, Sun Y, Hu C, Liu J, Gao A, Han H, Chai M, Zhang J, Zhou Y, Zhao Y (2020). Perivascular adipose tissue as an indication, contributor to, and therapeutic target for atherosclerosis. Front Physiol.

[21] Lin A, Dey D, Wong DTL, Nerlekar N (2019). Perivascular adipose tissue and coronary atherosclerosis: from Biology to Imaging phenotyping. Curr Atheroscler Rep.

[22] van Diemen PA, Bom MJ, Driessen RS, Schumacher SP, Everaars H, de Winter RW, van de Ven PM, Freiman M, Goshen L, Heijtel D (2021). Prognostic Value of RCA Pericoronary adipose tissue CT-Attenuation beyond high-risk plaques, plaque volume, and Ischemia. JACC Cardiovasc Imaging.

[23] Dong X, Li N, Zhu C, Wang Y, Shi K, Pan H, Wang S, Shi Z, Geng Y, Wang W (2023). Diagnosis of coronary artery disease in patients with type 2 diabetes mellitus based on computed tomography and pericoronary adipose tissue radiomics: a retrospective cross-sectional study. Cardiovasc Diabetol.

[24] Yu Y, Ding X, Yu L, Lan Z, Wang Y, Zhang J (2023). Prediction of microvascular complications in diabetic patients without obstructive coronary stenosis based on peri-coronary adipose tissue attenuation model. Eur Radiol.

[25] Ichikawa K, Miyoshi T, Osawa K, Nakashima M, Miki T, Nishihara T, Toda H, Yoshida M, Ito H (2022). High pericoronary adipose tissue attenuation on computed tomography angiography predicts cardiovascular events in patients with type 2 diabetes mellitus: post-hoc analysis from a prospective cohort study. Cardiovasc Diabetol.

[26] Ferket BS, Hunink MGM, Masharani U, Max W, Yeboah J, Burke GL, Fleischmann KE (2022). Lifetime Cardiovascular Disease risk by coronary artery calcium score in individuals with and without diabetes: an analysis from the multi-ethnic study of atherosclerosis. Diabetes Care.

[27] Sow MA, Magne J, Salle L, Nobecourt E, Preux PM, Aboyans V (2022). Prevalence, determinants and prognostic value of high coronary artery calcium score in asymptomatic patients with diabetes: a systematic review and meta-analysis. J Diabetes Complications.

[28] Orringer CE, Blaha MJ, Blankstein R, Budoff MJ, Goldberg RB, Gill EA, Maki KC, Mehta L, Jacobson TA (2021). The National Lipid Association scientific statement on coronary artery calcium scoring to guide preventive strategies for ASCVD risk reduction. J Clin Lipidol.

[29] Elnabawi YA, Oikonomou EK, Dey AK, Mancio J, Rodante JA, Aksentijevich M, Choi H, Keel A, Erb-Alvarez J, Teague HL (2019). Association of Biologic Therapy with Coronary Inflammation in patients with psoriasis as assessed by Perivascular Fat Attenuation Index. JAMA Cardiol.

[30] Angelini F, Bocchino PP, Imazio M (2021). Colchicine and coronary artery disease: a virtuous adoption. Eur Heart J.

[31] Malavazos AE, Meregalli C, Sorrentino F, Vignati A, Dubini C, Scravaglieri V, Basilico S, Boniardi F, Spagnolo P, Malagoli P (2023). Semaglutide therapy decreases epicardial fat inflammation and improves psoriasis severity in patients affected by abdominal obesity and type-2 diabetes. Endocrinol Diabetes Metab Case Rep.

[32] Blîndu E, Benedek I, Rodean I, Halațiu V, Raț N, Țolescu C, Mihăilă T, Roșca A, Mátyás B, Szabó E (2023). Regional differences in the Level of Inflammation between the Right and left coronary arteries– a Coronary computed Tomography Angiography Study of Epicardial Fat Attenuation Index in four scenarios of Cardiovascular emergencies. J Cardiovasc Emergencies.

